# One-pot synthesis of Au-M@SiO_2_ (M = Rh, Pd, Ir, Pt) core–shell nanoparticles as highly efficient catalysts for the reduction of 4-nitrophenol

**DOI:** 10.1038/s41598-022-11756-x

**Published:** 2022-05-10

**Authors:** Junfang Hao, Bin Liu, Shinya Maenosono, Jianhui Yang

**Affiliations:** 1 Key Laboratory of Synthetic and Natural Functional Molecule Chemistry of Ministry of Education, Shaanxi Key Laboratory of Physico-Inorganic Chemistry, College of Chemistry & Materials Science, Northwest University, Xi’an, 710069 People’s Republic of China; 2 School of Materials Science, Japan Advanced Institute of Science and Technology, 1-1 Asahidai, Nomi, Ishikawa 923-1292 Japan

**Keywords:** Chemistry, Materials science, Nanoscience and technology

## Abstract

The conversion of p-nitrophenol (4-NP) to p-aminophenol (4-AP) is of great significance for pharmaceutical and material manufacturing. In this work, Au-M@SiO_2_ (M = Rh, Pd, Ir, Pt) nanoparticles (NPs) with core–shell structures, which are expected to be excellent catalysts for the transformation of 4-NP to 4-AP, were synthesized by a facile one-pot one-step method. The structure and composition of the NPs were characterized through transmission electron microscopy, X-ray powder diffraction and X-ray photoelectron spectroscopy. Au-M@SiO_2_ (M = Rh, Pd, Ir, Pt) core–shell NPs showed excellent catalytic activity in the reduction of 4-NP, which is superior to most catalysts reported in the previous literature. The enhanced catalytic activity of Au-M@SiO_2_ core–shell NPs is presumably related to the bimetallic synergistic effect. This study provides a simple strategy to synthesize core–shell bimetallic NPs for catalytic applications.

## Introduction

P-nitrophenol (4-NP) is a common organic pollutant, while its reduction product (p-aminophenol, 4-AP) has a wide range of applications in pharmaceuticals, dyestuffs, and high polymer material fields^1–3^. Therefore, the reduction reaction of 4-NP to 4-AP is of vital practical importance. However, this reaction is very slow without catalyst in the presence of NaBH_4_^4^. In recent years, an increasing number of metal catalysts have been used for the reduction of 4-NP. Many investigations have focused on monometallic catalysts, such as Au, Ag, Pd, Cu, Ni and Co^5–10^. Compared to them, bimetallic catalysts exhibit better catalytic performance, such as Au–Pd alloy nanocrystals, silica (SiO_2_)-coated Ag/Au nanoparticles (NPs) and Pt–Rh alloyed nano-multipods^11–16^. These can be ascribed to their unique morphology and optimized electronic structure by synergy or alloy effects^17–22^. Au catalysts are widely employed in many fields, for example, CO oxidation, coupling reactions, oxidation of ethanol and degradation of 4-NP, because they are most active and selective under mild or even low-temperature conditions at the nanoscale^23–26^. Moreover, platinum-group metals (PGMs, such as Rh, Pd, Ir and Pt) have excellent chemical activity and thus exhibit advantageous performance^27–30^. According to previous reports, bimetallic Au-PGMs nanomaterials have attracted extensive interest for many catalytic applications, such as cyclization reactions, electrocatalysis and CO oxidation^31–34^. For example, Germano’s group reported ultrasmall Au@Pt core–shell nanostructures, which presented an enhanced performance for the hydrogen evolution reaction^35^. Mu’s group synthesized Au/Pd heterojunction@mesoporous SiO_2_ yolk-shell nanomaterials that exhibited high stability and plasmon-enhanced catalytic activity^36^. These results demonstrate that the combination of Au and PGMs is an effective way to enhance their catalytic performance. However, few reports have studied bimetallic Au-PGMs catalysts for the degradation of 4-NP. In addition, only bimetallic NPs tend to aggregate and leach during the reaction progress, resulting in decreased catalytic activity and stability^37,38^. Therefore, it would be a promising strategy to coat the surface of bimetallic Au-PGMs NPs with a SiO_2_ shell to overcome these problems.

In this study, we used a one-pot one-step method to synthesize Au-M@SiO_2_ (M = Rh, Pd, Ir, Pt) core–shell NPs. The stability of the catalyst was increased, and the agglomeration of NPs was prevented in the presence of the SiO_2_ shell. The morphology and structure of the Au-M@SiO_2_ core–shell NPs were determined by TEM, XRD and XPS. The catalytic performance of the Au-M@SiO_2_ (M = Rh, Pd, Ir, Pt) core–shell NPs was investigated in the model reaction of the reduction of 4-NP. This work provides a simplified way to prepare SiO_2_-coated bimetallic NP catalysts.

## Results and discussion

**Figure 1:**
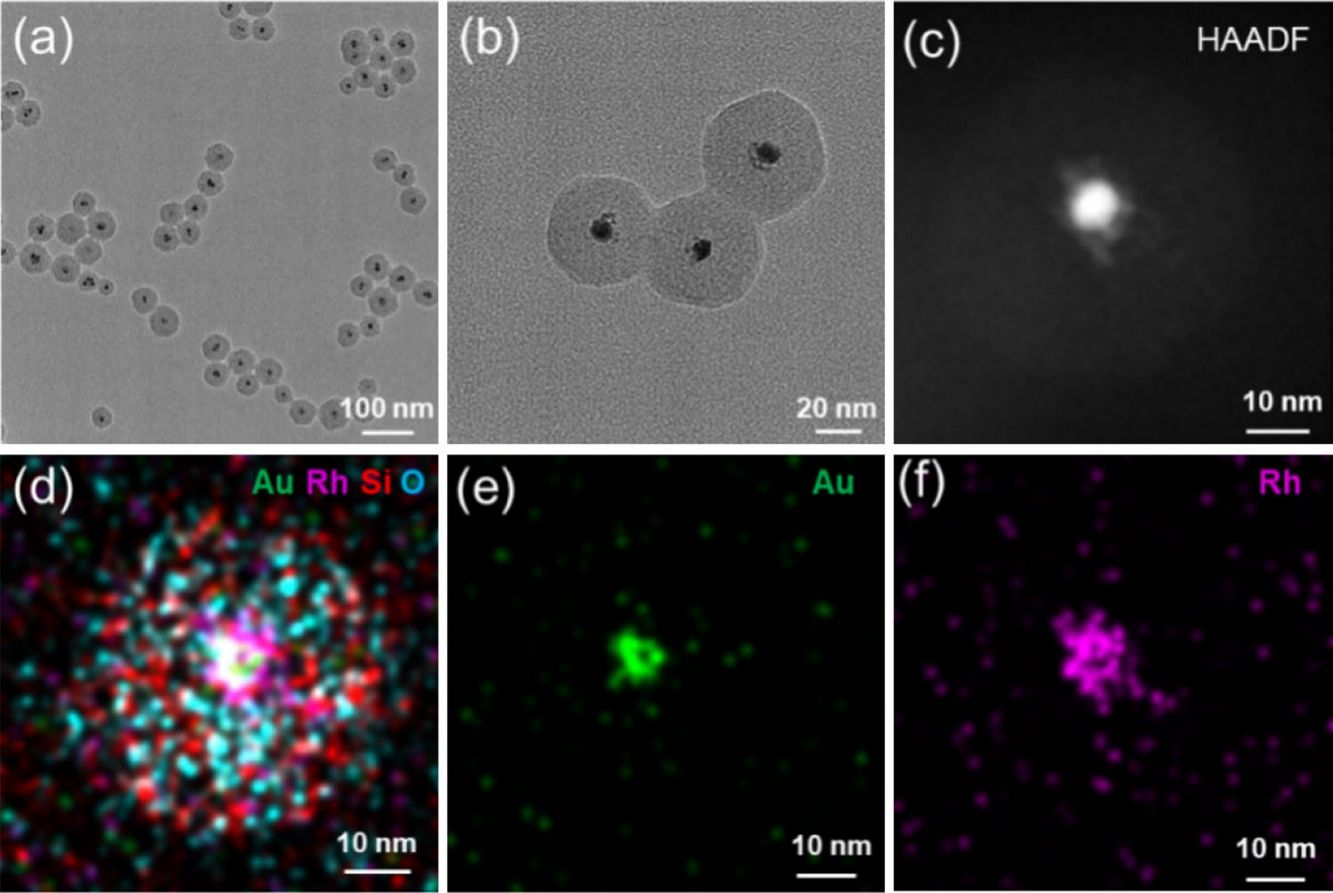
(**a**, **b**) TEM, (**c**) HAADF-STEM and (**d**-**f**) elemental mapping images of Au-Rh@SiO_2_ core–shell NPs.

Here, Au-Rh@SiO_2_ core–shell NPs are used as an example to illustrate the one-pot synthesis of silica-coated bimetallic Au-M (M = Rh, Pd, Ir and Pt) NPs. The morphology, structure and size of the samples were characterized by TEM. As shown in Fig. 1a,b, the as-prepared sample possesses core–shell structure and good monodispersity. The elemental mapping analysis of the sample in Fig. 1d–f demonstrates that Rh is distributed uniformly in the side of Au, and Au-Rh NPs were coated by silica shell, which further affirms the formation of core–shell structure. It is concluded that Au-Rh@SiO_2_ NPs with core–shell structures were synthesized successfully by this one-pot method. Au-Rh@SiO_2_ NPs have a core–shell structure. To further clarify the crystalline structure between Au and Rh, Au-Rh NPs synthesized without TEOS were characterized by TEM and high-resolution TEM. As shown in Fig. S1 in the ESM (Electronic Supplementary Material), Au-Rh NPs with core-satellite structure are similar to some Rh “planets” around an Au core. The formation of this unique structure is mainly because of the immiscibility and lattice mismatch of Au and Rh^39^. There are some heterojunction structures at the boundary between Au and Rh, which could contribute to their electron interaction and further improve their catalytic performance. The mass loading of Au-Rh in Au-Rh@SiO_2_ core–shell NPs by chemical method. It is assumed that the precursors HAuCl_4_, RhCl_3_ and TEOS are completely converted into Au, Rh and SiO_2_, respectively. The experimental mass loading of Au-Rh in Au-Rh@SiO_2_ core–shell NPs determined by their mass ratio is 7.9%. Au-Rh NPs was obtained by dissolving the silica shell with excess concentrated hydrofluoric acid. The experimental value is slightly smaller than the theoretical value (8.45%) because of the presence of CTAB in the silica layer.

**Figure 2:**
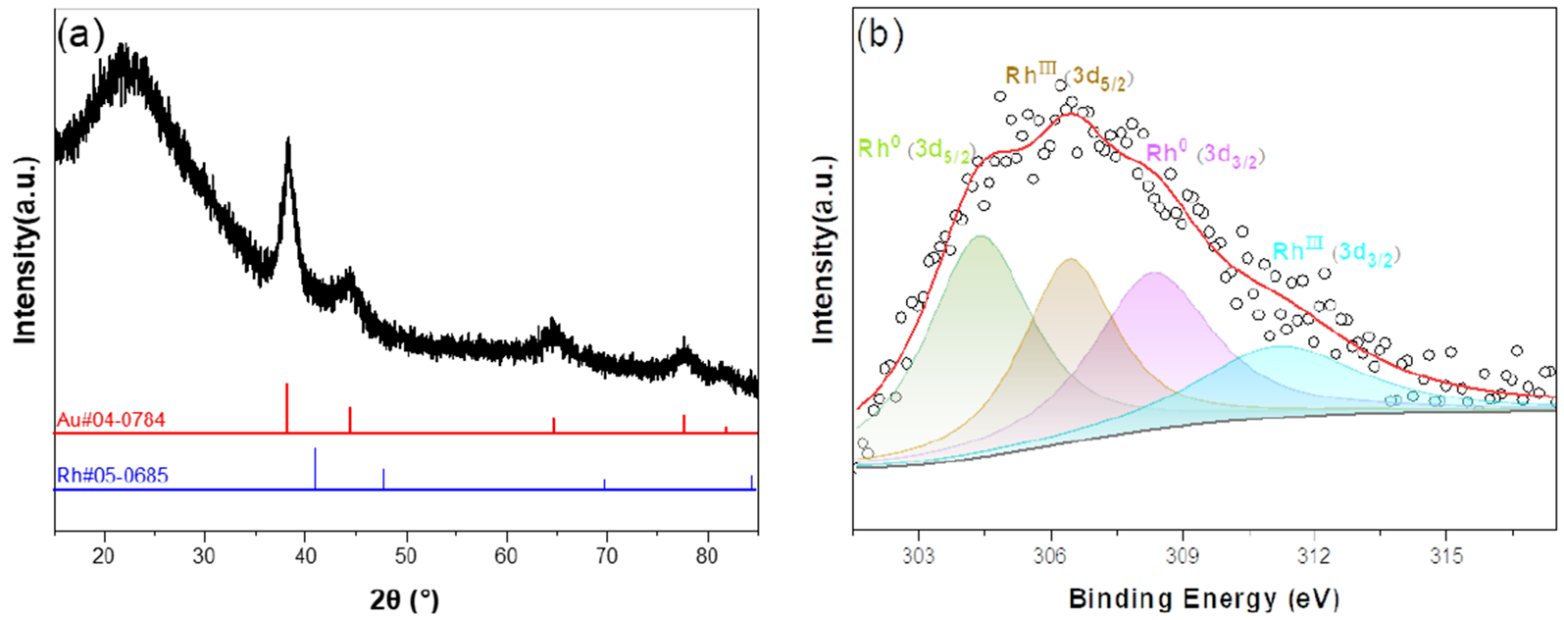
(**a**) XRD pattern and (**b**) Rh 3d spectrum of Au-Rh@SiO_2_ core–shell NPs.

The crystal structure of Au-Rh@SiO_2_ core–shell NPs was obtained by XRD. As shown in Fig. 2a, the main peaks at 38.6°, 45.0°, 65.2°, 78.1°, and 82.8° can be ascribed to the (111), (200), (220), (311), and (222) planes of Au (JCPDS: 04–0784)^40^. These peaks are in accordance with the peak positions of Au@SiO_2_ in Fig. S2, indicating the formation of a non-alloyed structure. However, there are no characteristic peaks of Rh. This might be attributed to the fact that the particle size of Rh is too small to be obtained. In addition, a broad peak stemming from the SiO_2_ shell is actually seen at around 2*θ* ≈ 20°. The elemental composition and states of Au-Rh@SiO_2_ core–shell NPs were further analyzed and characterized by XPS, as shown in Fig. 2b. The main peaks of the Rh 3d spectrum can be deconvoluted into four peaks. The two peaks at 304.6 eV and 308.2 eV can be related to Rh 3d_5/2_ and Rh 3d_3/2_ of Rh (0), and those with binding energies of 306.5 eV and 312.2 eV can be related to Rh 3d_5/2_ and Rh 3d_3/2_ of Rh (III)^41,42^. Compared with the standard values, the peaks of Rh species shift to higher binding energies^43^. Moreover, through peak area integral calculation, the ratio of Rh (0) and Rh (III) was found to be 1.45. The peak of Au 4f. is too weak to clearly observe in the survey-level XPS spectrum (Fig. S3), which is mainly because the Au-Rh “core-satellite” structure is coated by a silica shell^44^. This further confirmed the formation of a heterojunction structure rather than alloy, which is in agreement with the XRD result. The sample of Au-Rh NPs (Fig. S1) synthesized without TEOS was also characterized by XPS. As shown in Fig. S4, Au 4f. core-level and Rh 3d core-level signals are present due to the absence of a silica shell. The molar ratio of Au and Rh is 1.0 by the calculation of XPS peak areas, which is consistent with that of ICP-MS and the molar ratio of their precursors. In addition, the two peaks of Au (0) shift to the higher binding energy, which is mainly due to the electron interaction between Au and Rh^45^. The electronegativity of Au is higher than that of Rh, which results in an increased electron density of Au and a reduction of electron binding energy. Comparing the XPS spectra of Au-Rh@SiO_2_ core–shell and Au-Rh NPs, the ratio of Rh (0) and Rh (III) decreased from 1.45 to 0.75. These observations indicate that Rh NPs are oxidized^41,43^ or the alloy phase generates at the interface between Au and Rh during the XPS measurement^14–16^. This further revealed that the existence of silica shell could improve their stability.

**Figure 3:**
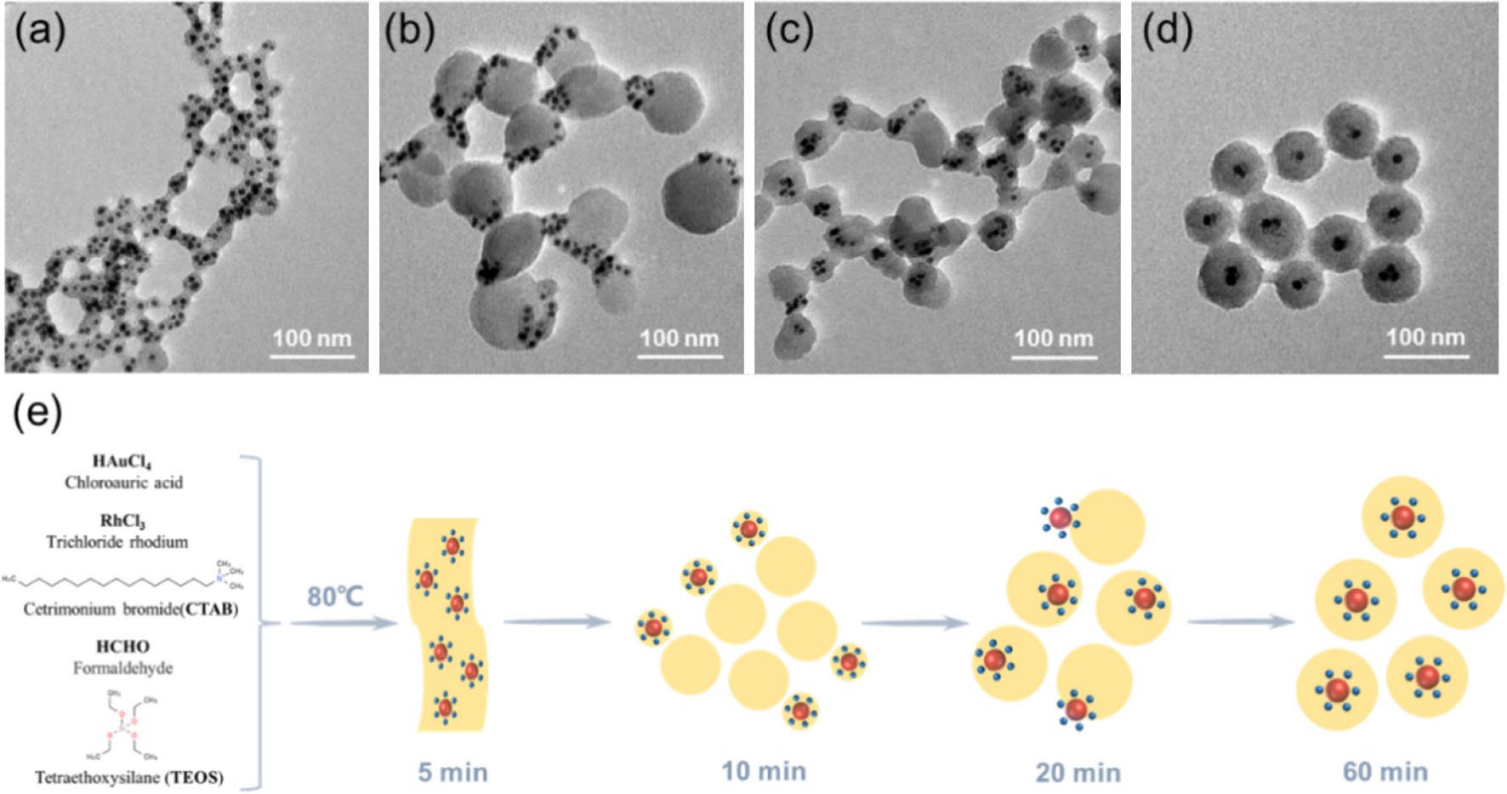
TEM images of the Au-Rh@SiO_2_ core–shell NPs at different reaction time: (**a**) 5 min, (**b**) 10 min, (**c**) 20 min and (**d**) 60 min. The forming process mechanism illustration of Au-Rh@SiO_2_ core–shell NPs (e).

The formation mechanism of Au-Rh@SiO_2_ core–shell NPs was investigated by acquiring TEM images of the samples taken at different reaction times. Here, Au-Rh@SiO_2_ core–shell NPs were synthesized by a one-pot one-step method developed by our research group and Zhao’s group^46,47^. HAuCl_4_ and RhCl_3_ were reduced by formaldehyde to result in the formation of Au-Rh core-satellite NPs after the reacted solution was heated at 80 °C for 5 min. At the same time, a thin layer of silica formed on the surface by base-catalyzed hydrolysis and polymerization of TEOS, as shown in Fig. 3a. In Fig. 3b, most of the Au-Rh NPs coated with a thin silica layer silica are on the outer edge of the silica spheres. The structures of the Au-Rh@SiO_2_ core–shell NPs initially formed after 20 min of reaction, as shown in Fig. 3c. However, Au-Rh NPs are not at the center of the silica spheres. At higher concentrations of CTAB, the lamellar micelles formed could induce anisotropic growth of SiO_2_ shells on the surface and Au-Rh NPs^48^. As the consumption of CTAB, the spherical micelles adopted may result in the isotropic growth of SiO_2_ shells. As shown in Fig. 3d, Au-Rh@SiO_2_ core–shell NPs finally formed at 60 min. The forming process mechanism of Au-Rh@SiO_2_ core–shell NPs is illustrated in Fig. 3e.

**Figure 4:**
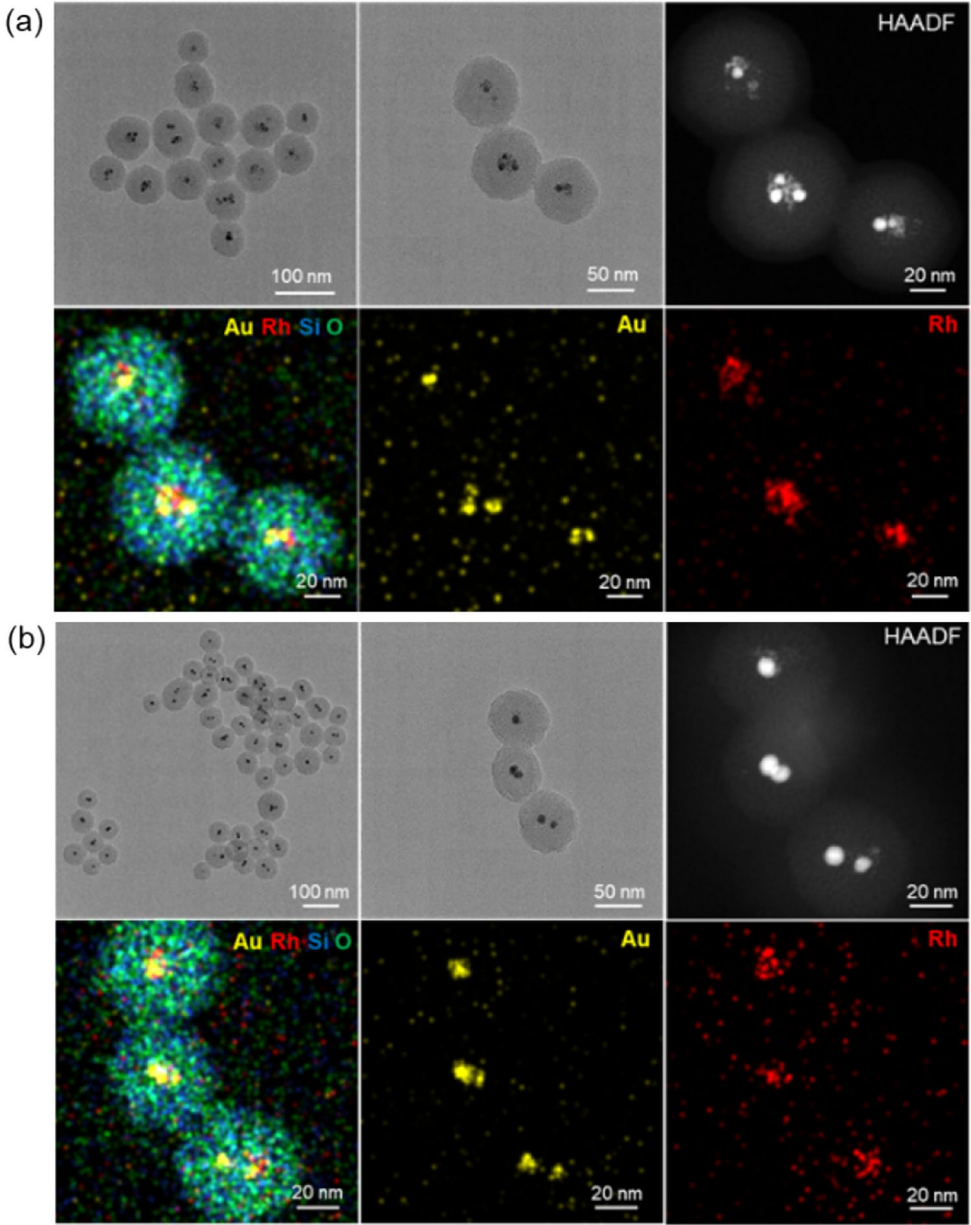
TEM images, HAADF-STEM image, and elemental mapping of (**a**) Au_1_-Rh_2_@SiO_2_ and (**b**) Au_2_-Rh_1_@SiO_2_ core–shell NPs, respectively.

We also investigated the effect of different precursor ratios (1:2 and 2:1) of Au and Rh on the resulting NPs. The samples were defined as Au_1_-Rh_2_@SiO_2_ and Au_2_-Rh_1_@SiO_2_ core–shell NPs, which were synthesized with HAuCl_4_ to RhCl_3_ molar ratios of 1:2 and 2:1, respectively. As demonstrated in Fig. 4, the two samples are with core–shell structures, the core is Au-Rh bimetallic NPs, and the shell with similar thickness is silica. When we carefully checked the structure of Au-Rh bimetallic NPs, it could be clearly seen that Au-Rh bimetallic NPs with core-satellite structure and isolated Rh NPs were encapsulated in the center of silica for the Au_1_-Rh_2_@SiO_2_ core–shell NPs in Fig. 4a. For Au_2_-Rh_1_@SiO_2_ core–shell NPs in Fig. 4b, Au is not coated well with Rh particles and Au-Rh core-satellite structure is not formed in the center of the silica shell. Similarly, we also examined the molar ratio of Au and Rh in Au_1_-Rh_2_ and Au_2_-Rh_1_ bimetallic NPs without silica shells synthesized in the absence of TEOS by XPS. As shown in Figs. S5 and S6, the molar ratios of Au to Rh of Au_1_-Rh_2_ and Au_2_-Rh_1_ bimetallic NPs are 0.56 and 1.68 according to the calculation of the corresponding XPS peak areas, respectively.

**Figure 5:**
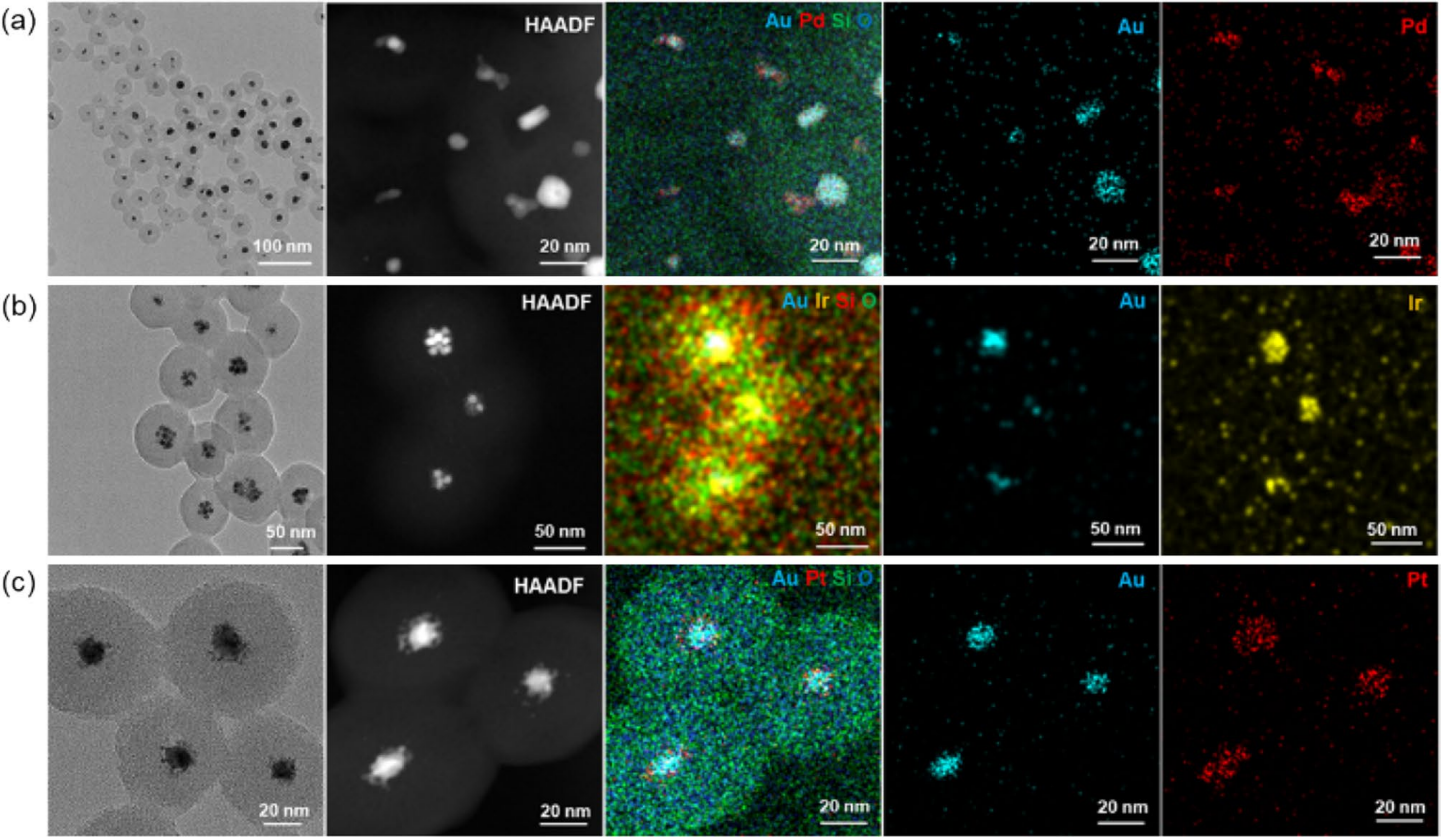
TEM image, HAADF-STEM image and elemental mapping of (**a**) Au-Pd@SiO_2_, (**b**) Au-Ir@SiO_2_ and (**c**) Au-Pt@SiO_2_ core–shell NPs, respectively.

We also employed this method to synthesize silica-coated bimetallic NPs consisting of Au and other platinum group metals (Pd, Ir, Pt). Figure 5a,b,c shows the TEM image, HAADF-STEM image and elemental mapping of Au-Pd@SiO_2_, Au-Ir@SiO_2_ and Au-Pt@SiO_2_ core–shell NPs, respectively. Combined with the results of TEM and XRD characterizations of Au-Pd, Au-Ir and Au-Pt bimetallic NPs shown in Figs. S7 and S8, Au-Pd@SiO_2_, Au-Ir@SiO_2_ and Au-Pt@SiO_2_ core–shell NPs can be successfully synthesized using this one-pot one-step method simply by changing the corresponding metallic precursor.

**Figure 6:**
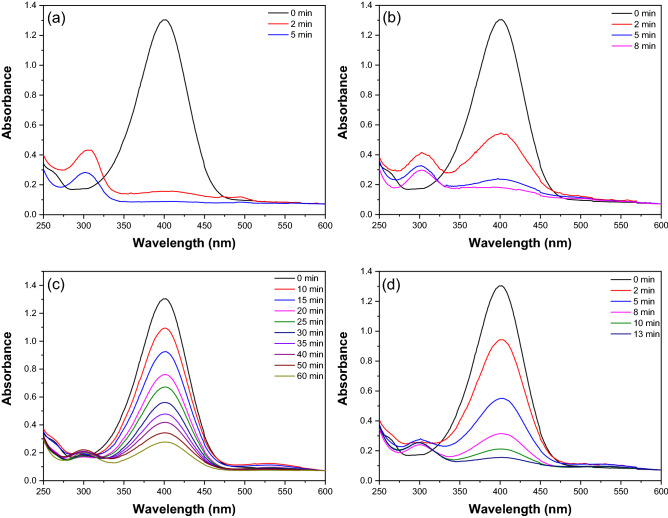
UV–vis spectra at different reaction times in the presence of Au-Rh@SiO_2_ core–shell NPs (**a**), Rh@SiO_2_ core–shell NPs (**b**), Au@SiO_2_ core–shell NPs (**c**) and the mixture of Rh@SiO_2_ and Au@SiO_2_ core–shell NPs (**d**), respectively.

To explore the catalytic reduction performance of 4-NP to 4-AP, UV–vis spectra in the presence of NaBH_4_ during different reaction times were monitored. The UV–vis absorption peaks of 4-NP and 4-AP are located at 400 nm and 300 nm in Fig. S9, respectively. The UV–vis spectra of Au-Rh@SiO_2_ core–shell NPs as a catalyst in Fig. 6a show that the reduction reaction of 4-NP finished within 5 min, indicating its good catalytic performance. The catalytic capability of Rh@SiO_2_ (Fig. S10a), Au@SiO_2_, and the mixture was further measured. Distinctly, in Fig. 6b–d, in the presence of Rh@SiO_2_, Au@SiO_2_ and the mixture as a catalyst, this reaction finished within 8 min, 60 min and 13 min, respectively. The broad peak at approximately 500–550 nm comes from the surface resonance plasmon resonance of Au NPs (Fig. S11). This phenomenon exhibits that Au-Rh@SiO_2_ core–shell NPs feature superior catalytic performance over their corresponding single component catalysts.

**Figure 7:**
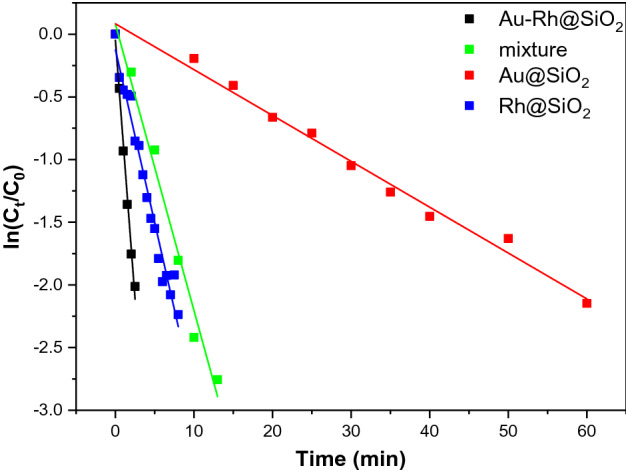
Plots of ln (C_t_/C_0_) versus time in the presence of Au-Rh@SiO_2_ core–shell NPs, Rh@SiO_2_ core–shell NPs, Au@SiO_2_ core–shell NPs and the mixture of Rh@SiO_2_ and Au@SiO_2_ core–shell NPs as catalysts.

In addition, the investigation on kinetics of the catalysts were further analyzed. If the ratio of C_t_/C_0_ (4-NP concentration at t minutes and 0 min) is proportional to time, this reduction reaction could be seen as first order kinetic, and the slope of the fitting line is a first order kinetic constant^49^. The corresponding results are shown in Fig. 7. The first-order kinetic constants of Au-Rh@SiO_2_ core–shell NPs, Rh@SiO_2_, Au@SiO_2_ and the mixture of Rh@SiO_2_ and Au@SiO_2_ NPs as catalysts are 0.826 min^−1^, 0.276 min^−1^, 0.037 min^−1^ and 0.228 min^−1^, respectively. Clearly, Au-Rh@SiO_2_ core–shell NPs have the maximum kinetic constant, also suggesting that this material exhibits the best catalytic performance. In addition, it can be observed from Table 1 that this catalyst is also superior to other bimetallic nanocatalysts reported in the previous literature^1,12,50–53^, revealing its excellent catalytic activity.

**Table 1:** Comparison of catalytic performance of bimetallic nanocatalysts for the reduction of 4-NP.

Catalyst	Mass	Kinetic constant	References
MOF-supported Au@Ag core–shell NPs	–	0.298 min^−1^	^12^
Au@Pd core–shell nanocubes	1 mL of 4.2 × 10^10^ particle/mL	2.05 ± 0.12 min^−1^	^50^
Ni–Pt NPs	1 mL of 0.1 mg/mL	0.116 min^−1^	^51^
Pd/Au(3:1)@g-C_3_N_4_-N	–	0.5310 min^−1^	^52^
Au/Pd core/shell NPs	75 μL of 2.0 mM	0.32 min^−1^	^1^
Cu/Ag alloy NPs	2 mg	0.237 min^−1^	^53^
Au-Rh@SiO_2_ core–shell structure	1 mL of 0.019 mg/mL	0.826 min^−1^	This work

**Figure 8:**
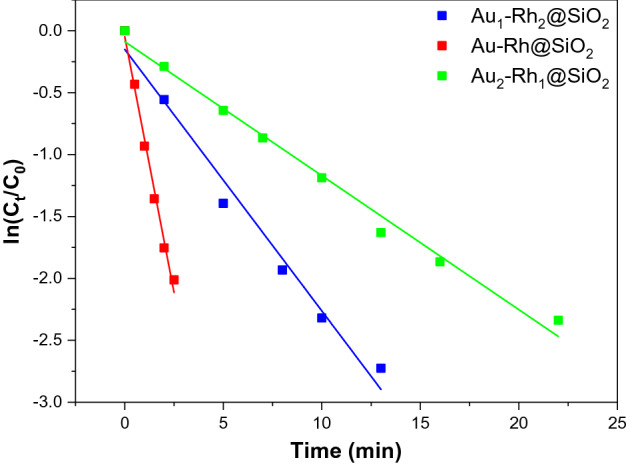
Plots of ln (C_t_/C_0_) versus time in the presence of Au_1_-Rh_2_@SiO_2_ core–shell NPs, Au-Rh@SiO_2_ core–shell NPs, Au_2_-Rh_1_@SiO_2_ core–shell NPs.

According to the results of TEM and XPS characterization of the Au-Rh@SiO_2_ core–shell NPs, Au-Rh bimetallic NPs exhibit core-satellite structures. There are some heterojunction structures at the boundary between Au and Rh, which result in their electron interaction and could improve their catalytic performance. To verify our hypothesis, the catalytic performance of Au-Rh@SiO_2_ core–shell NPs with different molar ratios of Au and Rh in Figs. S12 and S13 show UV–vis spectra at different reaction times in the presence of Au_1_-Rh_2_@SiO_2_ core–shell NPs and Au_2_-Rh_1_@SiO_2_ core–shell NPs. Figure 8 plots ln (C_t_/C_0_) versus time in the presence of Au-Rh@SiO_2_ core–shell NPs with different molar ratios of Au and Rh. Compared with the kinetic constant of Au-Rh@SiO_2_ core–shell NPs, Au_2_-Rh_1_@SiO_2_ core–shell NPs exhibited the lowest catalytic performance due to the low content of Rh. However, for the sample of Au_1_-Rh_2_@SiO_2_ core–shell NPs, its catalytic performance was still lower than that of Au-Rh@SiO_2_ core–shell NPs. As observed in Fig. 3a, a thicker layer of Rh NPs was formed and prevented the synergistic effect of Au and Rh. In conclusion, the Au-Rh@SiO_2_ core–shell NPs as highly efficient catalysts for the reduction of 4-NP could be attributed to the synergistic effect coming from their unique core–shell structure and electronic interaction of Au and Rh.

**Figure 9:**
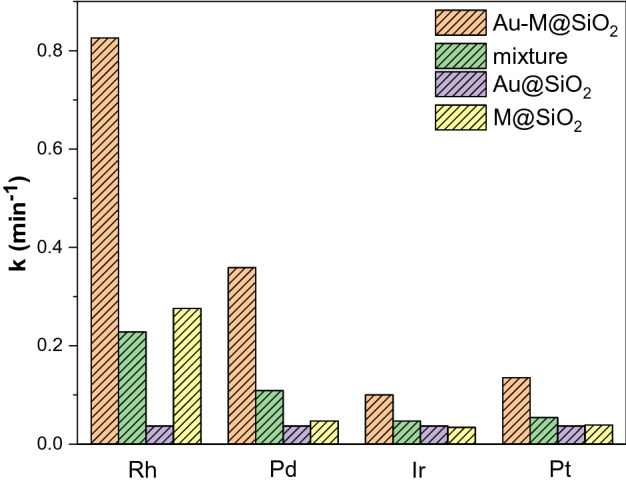
The catalytic performance of Au-M@SiO_2_ (M = Rh, Pd, Ir, Pt) core–shell NPs for reduction of 4-NP.

The synergistic effect was also confirmed by other silica-coated Au-based PGMs bimetallic NPs. Figs. S14, S15, S16 and S17 show the catalytic performance of Au-M@SiO_2_ (M = Pd, Ir, Pt) core–shell NPs for the reduction of 4-NP. They exhibit highly efficient catalytic activity with first-order kinetic constants of 0.359 min^−1^, 0.100 min^−1^ and 0.135 min^−1^, respectively. As shown in Fig. 9, we summarized the Au-M@SiO_2_ (M = Rh, Pd, Ir, Pt) core–shell NPs as catalysts for the reduction of 4-NP. Au-Rh@SiO_2_ core–shell NPs exhibit the highest catalytic activity for the reduction of 4-NP in the excess of NaBH_4,_ which is due to the higher adsorption energy of 4-NP on Rh surfaces and more efficient interfacial electron transfer between Au and Rh.

**Figure 10:**
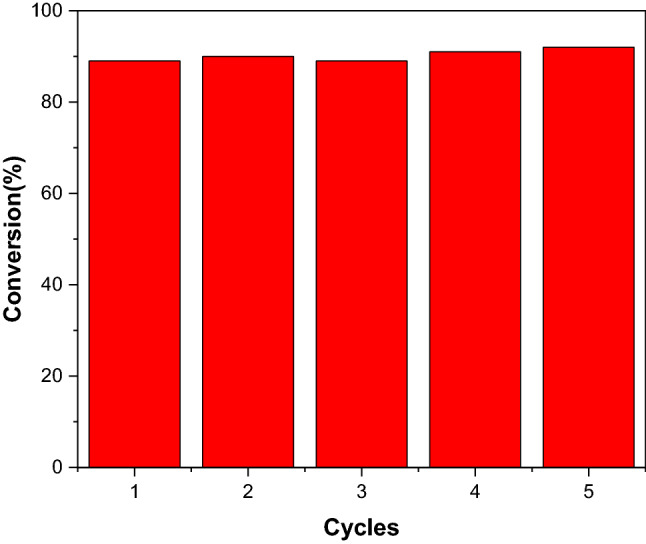
The conversion rate for different cycles of Au-Rh@SiO_2_ core–shell NPs for reduction of 4-NP.

The recyclability of Au-Rh@SiO_2_ core–shell NPs shown in Fig. 10 demonstrates that the conversion rate is still around 90% after 5 cycles, indicating its high stability. As shown in Fig. S18, the first-order kinetic constant of Au-Rh NPs to the reduction reaction is 0.807 min^−1^, which is slightly lower than that (0.826 min^−1^) of Au-Rh@SiO_2_ core–shell NPs. As observed in Fig. S19, Au-Rh bimetallic NPs occurs obvious agglomeration after catalysis three times. The conversion rate of Au-Rh bimetallic NPs after catalysis three times reduced to 79%. On the contrary, Au-Rh@SiO_2_ core–shell NPs still can keep the original morphology and structure after five cycles of catalysis. It illustrates that SiO_2_ shell layers can improve the stability.

Therefore, the highly efficient catalytic performance of Au-Rh@SiO_2_ core–shell NPs mainly comes from the synergistic effect of Au-Rh and core–shell structure. The catalytic performance of Au-Rh@SiO_2_ core–shell NPs is superior to that of the corresponding monometallic@SiO_2_ NPs or their mixture. TEM and XPS characterizations indicate the formation of heterojunction structures at the boundary of Au-Rh and their electron interaction. The electron interaction is insufficient as decreasing the molar ratio of Rh/Au and is hindered as increasing the molar ratio of Rh/Au. It sufficiently demonstrates the synergistic effect of Au-Rh results in the highly efficient catalytic performance for the reduction of 4-NP. The SiO_2_ shell layer improves their high catalytic cycle stability and recovery.

## Conclusions

In summary, Au-M@SiO_2_ (M = Rh, Pd, Ir, Pt) NPs with core–shell structures were successfully synthesized by a facile one-pot one-step method. The morphology, crystal structure, elemental composition and formation mechanism of these core–shell NPs were investigated. Au-M@SiO_2_ (M = Rh, Pd, Ir, Pt) core–shell NPs showed excellent catalytic activity in the reduction of 4-NP, which is superior to most catalysts reported in the previous literature. The enhanced catalytic activity of Au-M@SiO_2_ core–shell NPs is presumably related to the bimetallic synergistic effect. This study provides a simple strategy to synthesize core–shell bimetallic NPs for catalytic applications.

## Methods

### Materials

Chloroauric acid tetrahydrate (HAuCl_4_·4H_2_O, ˃ 47.0%, Beijing Huawei Ruike Chemical Co., Ltd), palladium chloride acid (H_2_PdCl_4_, > 97.0%, Shanghai Macklin Biochemical Co., Ltd), chloroplatinic acid hexahydrate (H_2_PtCl_6_·6H_2_O, ≥ 37.0%, Shanghai Institute of Fine Chemical Materials), rhodium chloride trihydrate (RhCl_3_·3H_2_O, ≥ 39.0%, Xi'an Kaili Chemical Co., Ltd), cetyltrimethylammonium bromide (CTAB, C_19_H_42_BrN, 97.0%, Shanghai Macklin Biochemical Co., Ltd), iridium trichloride (IrCl_3_, ≥ 42.5%, Shanghai Bide Pharmaceutical Technology Co., Ltd), sodium hydroxide (NaOH, ≥ 96.0%, Tianjin Tianli Chemical Reagent Co., Ltd), formaldehyde solution (HCHO, 37.0%-40.0%, Tianjin Fuyu Chemical Reagent Co., Ltd), 4-NP (C_6_H_5_NO_3_, 99%, Damas-beta Reagent Co., Ltd), sodium borohydride (NaBH_4_, Tianjin Cameo Chemical Reagent Co., Ltd.) and tetraethyl orthosilicate (TEOS, Tianjin Cameo Chemical Reagent Co., Ltd.) were directly used without further purification. Anhydrous alcohol and deionized water were used in all experiments.

### Synthesis

Au@SiO_2_ core–shell NPs were synthesized by a facile one-pot one-step method using formaldehyde as a reducing agent^46,47^. Similarly, M@SiO_2_ (M = Rh, Pd, Ir and Pt) core–shell NPs were prepared by changing the corresponding metal precursors.

The above method is still suitable for the synthesis of bimetallic Au-M@SiO_2_ (M = Rh, Pd, Ir and Pt) core–shell NPs. Taking Au-Rh@SiO_2_ core–shell NPs as an example, in a typical synthesis, 38 mL of deionized water, 6 mL of ethanol, 0.07 g of CTAB, 1 mL of HCHO, 1 mL of NaOH (0.5 mol/L), 1 mL of 8.14 mmol/L HAuCl_4_, 1 mL of 8.14 mmol/L RhCl_3_ and 0.10 mL of TEOS were mixed in a 100 mL of beaker under ultrasonication. The beaker was left in an oil bath at 80 °C for 60 min under stirring. After cooling to room temperature naturally, the product was centrifuged at 11,000 rpm for 20 min and washed two times with water to remove the surfactant and unreacted chemicals. Finally, the precipitate was dissolved in 3 mL of water for further characterization.

Au-Rh@SiO_2_ core–shell NPs with different Au/Rh molar ratios were obtained under the same reaction conditions by changing the molar ratio of HAuCl_4_ to RhCl_3_ to 1:2 and 2:1, respectively.

The other Au-M@SiO_2_ (M = Pd, Ir and Pt) core–shell NPs were prepared by replacing RhCl_3_ with H_2_PdCl_4_, RhCl_3_ and H_2_PtCl_6_, respectively.

The procedure of preparing Au-M (M = Rh, Pd, Ir and Pt) bimetallic NPs is similar to the typical synthesis of Au-Rh@SiO_2_ core–shell NPs in the absence of TEOS.

### Characterization

Transmission electron microscopy (TEM) images were obtained from Talos F200X (FEI, 200 kV) equipped with a high-angle annular dark field (HAADF) detector and an energy dispersive X-ray (EDX) spectrometer. The TEM samples were prepared by dropping nanoparticle dispersions onto 200-mesh copper grids covered with carbon membranes. X-ray diffraction (XRD) measurements were carried out on a Bruker D8 Advance diffractometer with Cu Kα radiation (λ = 1.54059 Å). X-ray photoelectron spectrophotometer (XPS) spectra were taken by using a PHI5000 Versa Probe III spectrometer with an excitation source of Al Kα. The samples for XRD and XPS were prepared by dropping nanoparticle dispersion onto silicon wafers and dried naturally at room temperature. All spectra were recalibrated with respect to the C 1 s core level peak at 284.8 eV of carbon contamination. UV–vis absorption spectra were obtained on an ultraviolet–visible spectrophotometer (SP-75). The element ratio was determined by inductively coupled plasma-mass spectrometry (ICP-MS, Agilent 7900).

### Catalytic reduction of 4-nitrophenol

1 mL of freshly prepared NaBH_4_ (0.06 mol/L) was mixed with 1 mL of 4-NP (1.7 × 10^–4^ mol/L) in a quartz cuvette with 1 cm path length. The detailed illustration takes Au-Rh@SiO_2_ NPs as an example. 1 mL of Au-M@SiO_2_ core–shell NP aqueous dispersion was quickly added to the above solution. The catalytic reaction progress of the conversion of 4-NP to 4-AP was observed via UV–vis spectroscopy between 250 and 600 nm at ambient temperature.

## Supplementary Information

Supplementary Information.

## Data Availability

The datasets used and/or analysed during the current study available from the corresponding author on reasonable request. All data generated or analysed during this study are included in this published article and its supplementary information files.
