# Supplementary material for: One-pot synthesis of Au-M@SiO2 (M = Rh, Pd, Ir, Pt) core–shell nanoparticles as highly efficient catalysts for the reduction of 4-nitrophenol
Source: Sci Rep. 2022 May 10;12:7615. doi: 10.1038/s41598-022-11756-x (PMC9091199; doi:10.1038/s41598-022-11756-x)
Supplement: Supplementary file 1 — Supplementary Information. [file 41598_2022_11756_MOESM1_ESM.pdf]

Electronic supplementary material

One-pot synthesis of Au-M@SiO_2 (M = Rh, Pd, Ir, Pt) core-shell nanoparticles as highly efficient catalysts for the reduction of 4-nitrophenol

Junfang Hao[1], Bin Liu[1], Shinya Maenosono[2]*, and Jianhui Yang[1]*

[1]Key Laboratory of Synthetic and Natural Functional Molecule Chemistry of Ministry of Education, Shaanxi Key Laboratory of Physico-Inorganic Chemistry, College of Chemistry & Materials Science, Northwest University, Xi'an 710069, P. R. China.
[2]School of Materials Science, Japan Advanced Institute of Science and Technology, 1-1 Asahidai, Nomi, Ishikawa 923-1292, Japan.
*Corresponding. shinya@jaist.ac.jp and jianhui@nwu.edu.cn

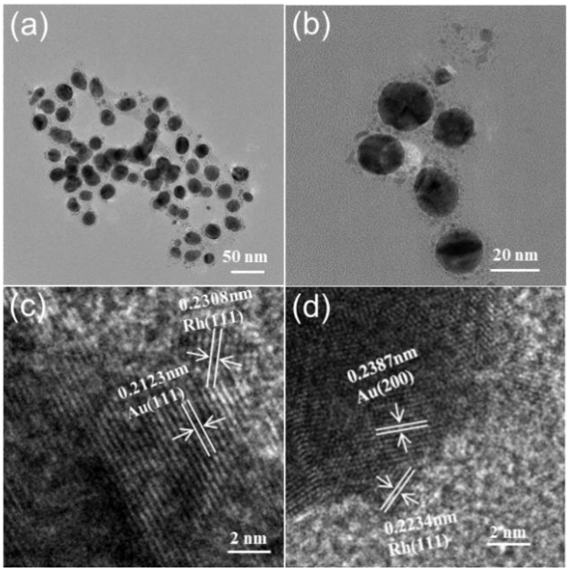

Figure S1. (a-b) TEM images at different scale and (c-d) HRTEM images of Au-Rh NPs synthesized without TEOS.

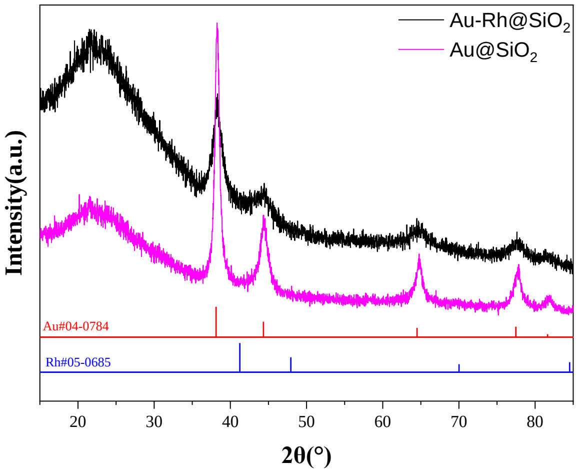

Figure S2. XRD patterns of Au-Rh@SiO_2 and Au@SiO_2 core-shell NPs.

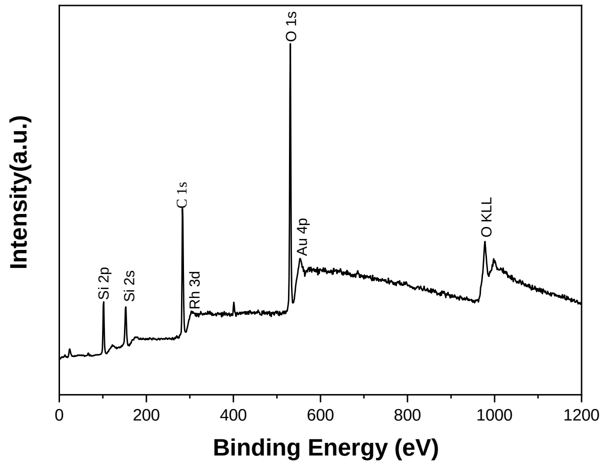

Figure S3. Survey-level XPS spectrum of Au-Rh@SiO_2 core-shell NPs.

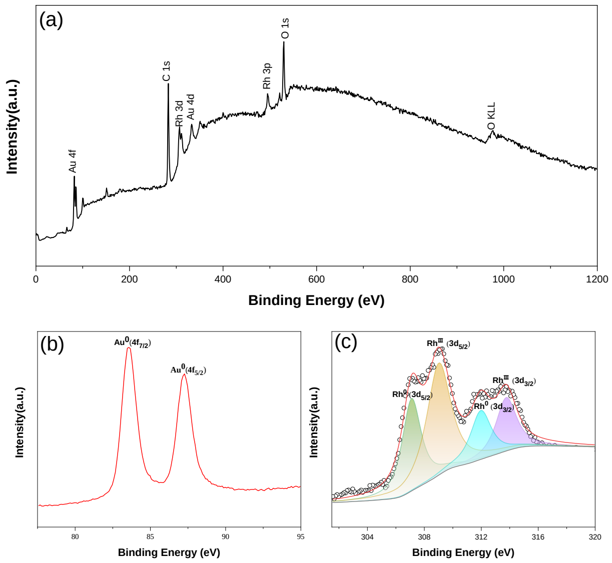

Figure S4. (a) Survey-level XPS spectrum, (b) Au 4f core-level and (c) Rh 3d core-level spectra of Au-Rh (1:1) NPs synthesized without TEOS.

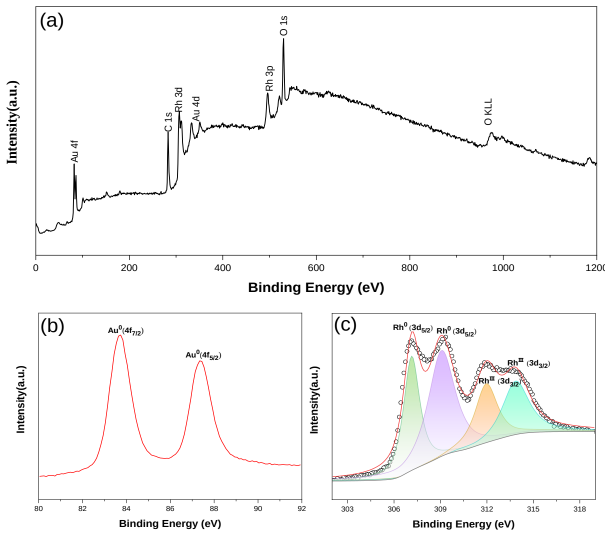

Figure S5. (a) Survey-level XPS spectrum (b) Au 4f core-level and (c) Rh 3d core-level spectra of Au-Rh (1:2) NPs.

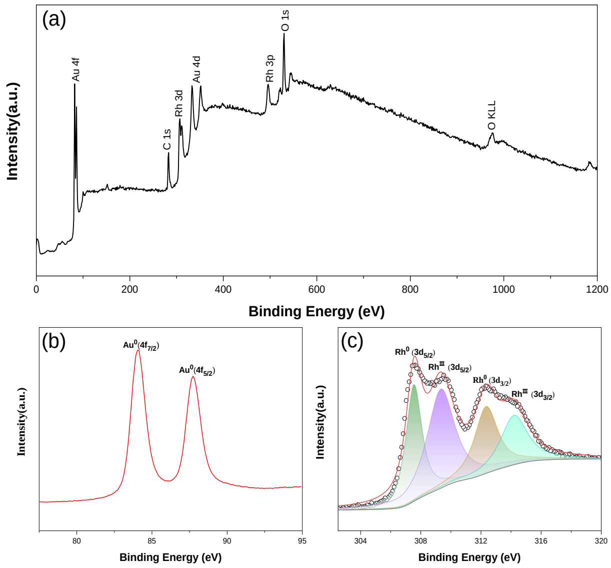

Figure S6. (a) Survey-level XPS spectrum (b) Au 4f core-level and (c) Rh 3d core-level spectra of Au-Rh (2:1) NPs.

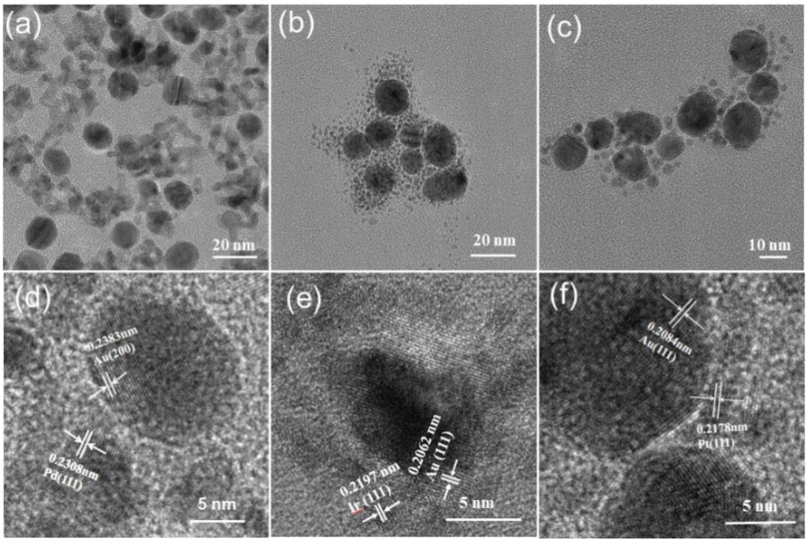

Figure S7. (a-c) TEM images and (d-f) HR-TEM images of Au-Pd, Au-Ir and Au-Pt bimetallic NPs, respectively.

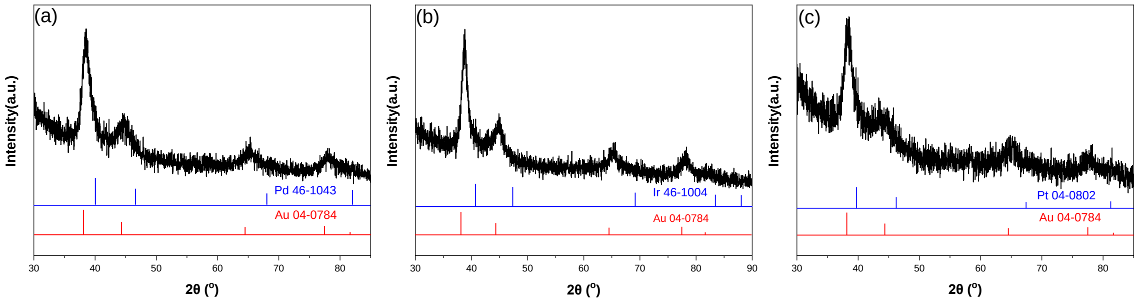

Figure S8. XRD patterns of (a) Au-Pd, (b) Au-Ir and (c) Au-Pt bimetallic NPs, respectively.

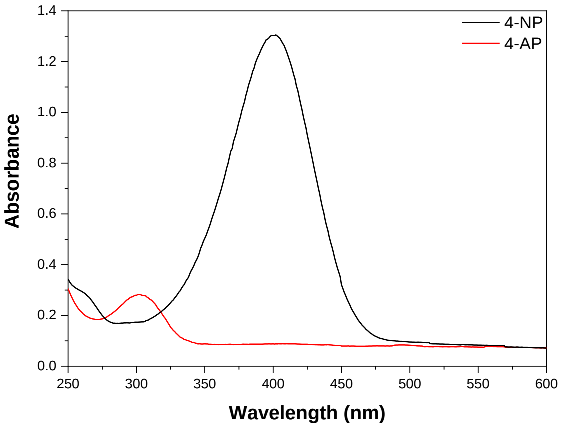

Figure S9. Uv-vis absorption spectra of 4-NP and 4-AP.

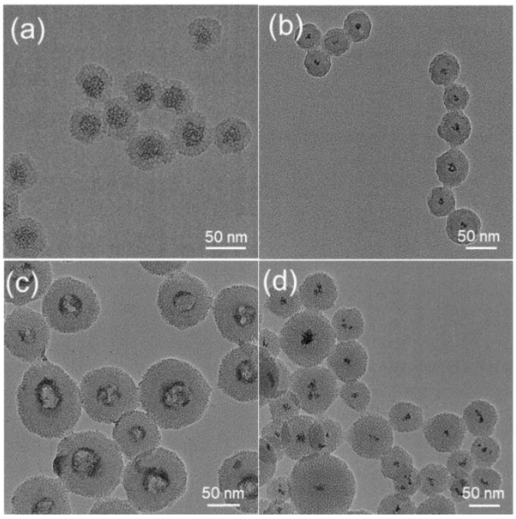

Figure S10. TEM images of (a) $Rh@SiO_2$, (b) $Pd@SiO_2$, (c) $Ir@SiO_2$ and (d) $Pt@SiO_2$ core-shell NPs.

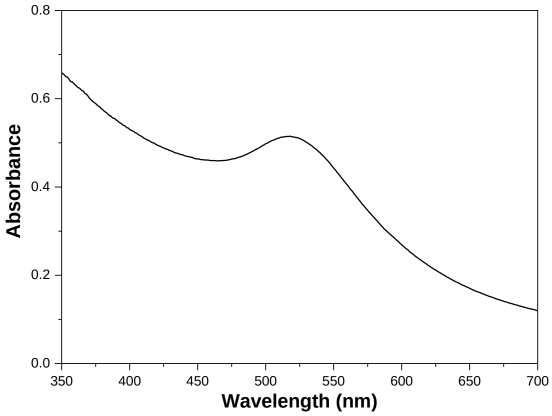

Figure S11. Uv-vis absorption spectrum of $Au\text{-}Rh@SiO_2$ core-shell NPs aqueous.

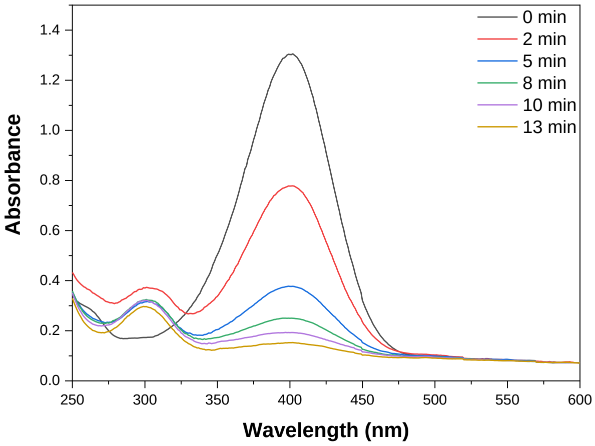

Figure S12. Uv-vis spectra at different reaction times in the presence of $Au_1\text{-}Rh_2@SiO_2$ core-shell NPs.

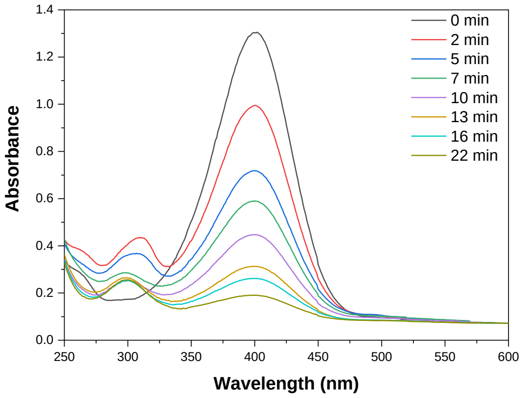

Figure S13. Uv-vis spectra at different reaction times in the presence of Au_2-Rh_1@SiO_2 core-shell NPs.

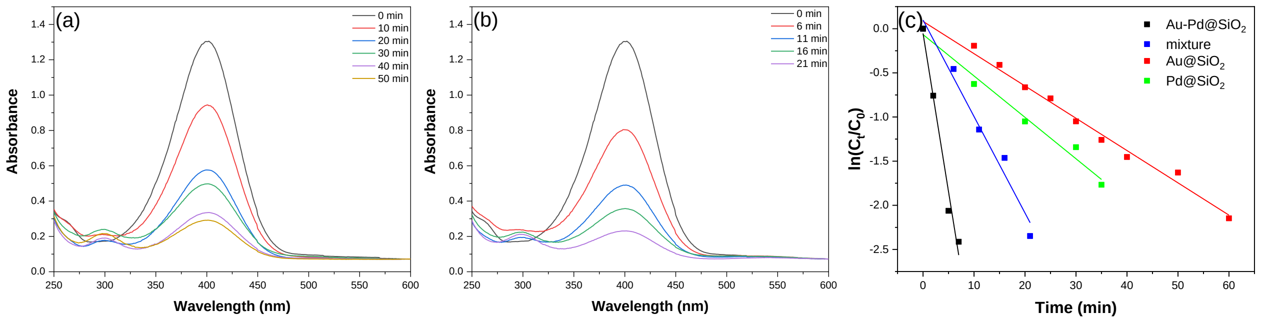

Figure S14. (a) Uv-vis spectra at different reaction times of Pd@SiO_2 core-shell NPs, (b) mixture of 1/2 Pd@SiO_2 and 1/2 Au@SiO_2 core-shell NPs, and (c) plots of ln (C_t/C_0) vs. time in the presence of different type catalysts.

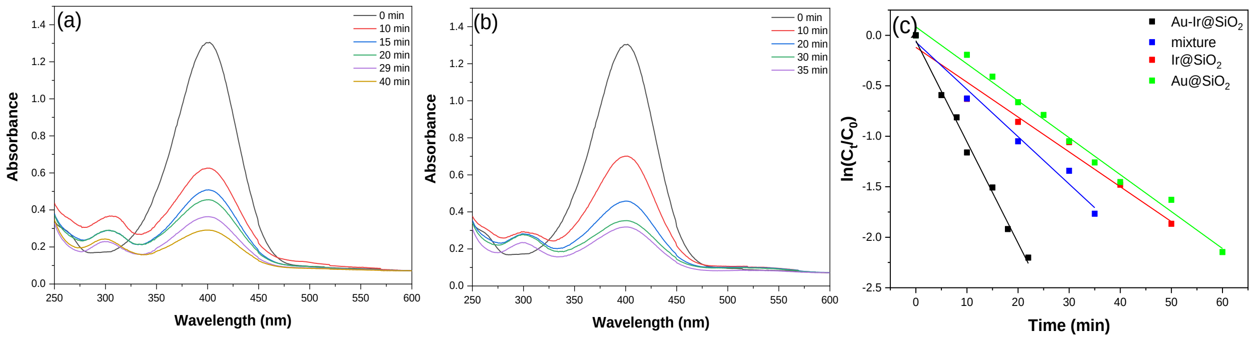

Figure S15. (a) Uv-vis spectra at different reaction times of Ir@SiO_2 core-shell NPs, (b) mixture of 1/2 Ir@SiO_2 and 1/2 Au@SiO_2 core-shell NPs, and (c) plots of ln (C_t/C_0) vs. time in the presence of different type catalysts.

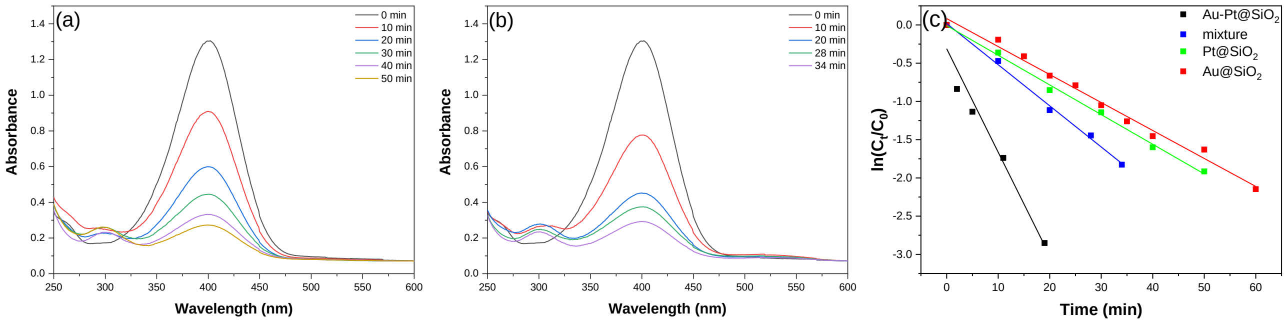

Figure S16. (a) Uv-vis spectra at different reaction times of Pt@SiO_2 core-shell NPs, (b) mixture of 1/2 Pt@SiO_2 and 1/2 Au@SiO_2 core-shell NPs, and (c) plots of ln (C_t/C_0) vs. time in the presence of different type catalysts.

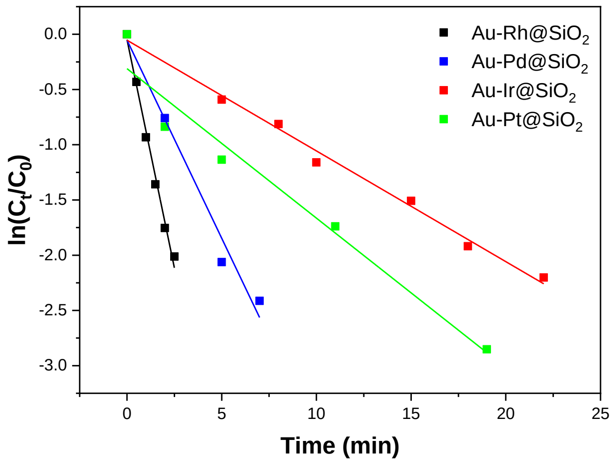

Figure S17. The plots of ln (C_t/C_0) vs. time in the presence of Au-Rh@SiO_2, Au-Pd@SiO_2, Au-Ir@SiO_2 and Au-Pt@SiO_2 core-shell NPs as catalysts for the reduction of 4-NP, respectively.

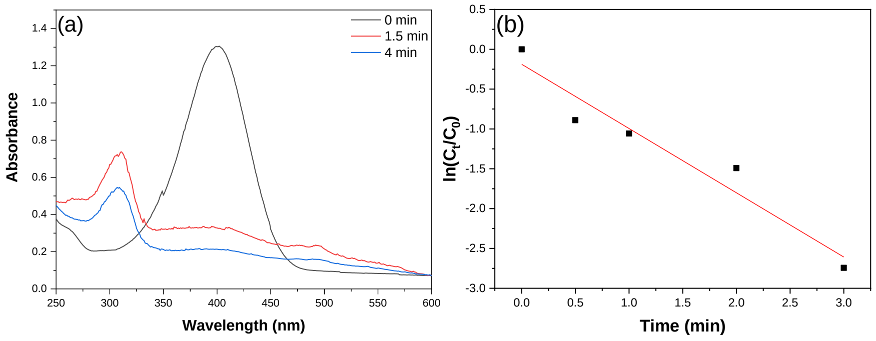

Figure S18. (a) UV-vis spectra at different reaction times and (b) plot of ln (C_t/C_0) vs. time in the presence of Au-Rh NPs.

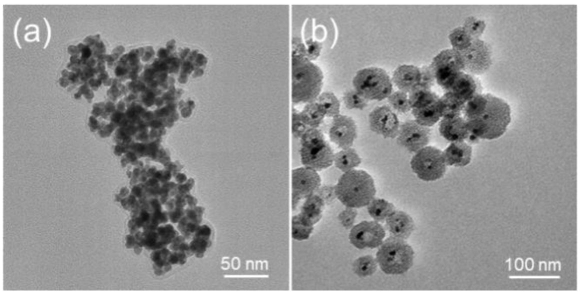

Figure S19. TEM images of (a) Au-Rh bimetallic NPs after catalysis three times and (b) Au-Rh@SiO_2 core-shell NPs after catalysis five times.
